# Integrase inhibitor (INI) genotypic resistance in treatment-naive and raltegravir-experienced patients infected with diverse HIV-1 clades

**DOI:** 10.1093/jac/dkv243

**Published:** 2015-08-26

**Authors:** Tomas Doyle, David T. Dunn, Francesca Ceccherini-Silberstein, Carmen De Mendoza, Frederico Garcia, Erasmus Smit, Esther Fearnhill, Anne-Genevieve Marcelin, Javier Martinez-Picado, Rolf Kaiser, Anna Maria Geretti

**Affiliations:** 1 Department of Infectious Diseases, King's College London, London, UK; 2 MRC Clinical Trial Unit at UCL, London, UK; 3 Faculty of Medicine and Surgery, University of Rome Tor Vergata, Rome, Italy; 4 Research Institute and Hospital Puerta de Hierro, Madrid, Spain; 5 HU San Cecilio, Granada, Spain; 6 Heart of England NHS Foundation Trust, Birmingham, UK; 7 AP-HP, Hôpital Pitié-Salpêtrière, INSERM-Sorbonne Universités, UPMC Univ Paris 06, UMR_S 1136, Paris, France; 8 IrsiCaixa, ICREA and UVic-UCC, Barcelona, Spain; 9 Institute of Virology, University of Cologne, Cologne, Germany; 10 Department of Clinical Infection, Microbiology and Immunology, Institute of Infection and Global Health, University of Liverpool, Liverpool, UK

## Abstract

**Objectives:**

The aim of this study was to characterize the prevalence and patterns of genotypic integrase inhibitor (INI) resistance in relation to HIV-1 clade.

**Methods:**

The cohort comprised 533 INI-naive subjects and 255 raltegravir recipients with viraemia who underwent integrase sequencing in routine care across Europe, including 134/533 (25.1%) and 46/255 (18.0%), respectively, with non-B clades (A, C, D, F, G, CRF01, CRF02, other CRFs, complex).

**Results:**

No major INI resistance-associated mutations (RAMs) occurred in INI-naive subjects. Among raltegravir recipients with viraemia (median 3523 HIV-1 RNA copies/mL), 113/255 (44.3%) had one or more major INI RAMs, most commonly N155H (45/255, 17.6%), Q148H/R/K + G140S/A (35/255, 13.7%) and Y143R/C/H (12/255, 4.7%). In addition, four (1.6%) raltegravir recipients showed novel mutations at recognized resistance sites (E92A, S147I, N155D, N155Q) and novel mutations at other integrase positions that were statistically associated with raltegravir exposure (K159Q/R, I161L/M/T/V, E170A/G). Comparing subtype B with non-B clades, Q148H/R/K occurred in 42/209 (20.1%) versus 2/46 (4.3%) subjects (*P* = 0.009) and G140S/A occurred in 36/209 (17.2%) versus 1/46 (2.2%) subjects (*P* = 0.005). Intermediate- to high-level cross-resistance to twice-daily dolutegravir was predicted in 40/255 (15.7%) subjects, more commonly in subtype B versus non-B clades (39/209, 18.7% versus 1/46, 2.2%; *P* = 0.003). A glycine (G) to serine (S) substitution at integrase position 140 required one nucleotide change in subtype B and two nucleotide changes in all non-B clades.

**Conclusions:**

No major INI resistance mutations occurred in INI-naive subjects. Reduced occurrence of Q148H/R/K + G140S/A was seen in non-B clades versus subtype B, and was explained by the higher genetic barrier to the G140S mutation observed in all non-B clades analysed.

## Introduction

Raltegravir was the first integrase inhibitor (INI) to receive approval for the treatment of HIV infection, marking an important therapeutic advance through the provision of a novel class of antiretroviral agents. Raltegravir inhibits the HIV integrase enzyme at the final integration step, the strand transfer reaction, which otherwise results in the covalent linkage of host and viral DNA.^1^ Raltegravir has shown efficacy in clinical trials of ART-naive and ART-experienced patients.^2,3^ However, raltegravir possesses a low to moderate genetic barrier to resistance and single amino acid substitutions in integrase are sufficient to compromise activity.^4,5^ Previously, the largest series of INI resistance data came from the Phase 3 BENCHMARK 1 and 2 trials, which recruited ART-experienced patients infected primarily with HIV-1 subtype B.^3,6^ Overall, 105/462 (23%) patients receiving raltegravir with an optimized background regimen experienced virological failure at week 48, of whom 68% had evidence of raltegravir resistance.^3,6^ Three genotypic resistance pathways were identified: N155H (±E92Q), Q148H/R/K (±G140S) and Y143R (±T97A). A survey of genotypic resistance tests performed through routine care in a referral laboratory in the USA has shown similar integrase mutational profiles, and whereas the study population had unknown ART status and carried nearly uniformly HIV-1 subtype B,^7^ a more recent study has shown consistent findings in a large INI-experienced cohort.^8^

Two further INIs, elvitegravir and dolutegravir, have recently been approved. Elvitegravir has a similar genetic barrier to and extensive cross-resistance with raltegravir.^9–12^ *In vitro*, the activity of dolutegravir is not compromised by mutations occurring at integrase codon 143 or 155 in isolation, although whether mutations can affect susceptibility in combination with multiple others is the subject of ongoing research. Q148 mutations in combination with other mutations (typically at codon L74, E138 or G140) reduce dolutegravir *in vitro* susceptibility by 10- to 20-fold.^13–16^ Findings from clinical studies are consistent with the *in vitro* susceptibility data, and indicate that the Q148 mutation pathway significantly markedly affects dolutegravir activity among INI-experienced patients, even when the drug is used twice daily.^17–23^

Data on the genotypic pathways of INI resistance occurring with HIV-1 clades other than subtype B are limited to small cohorts. These studies have suggested that there may be important differences in the resistance profiles of subtype C, CRF01_AE and CRF02_AG, including a high prevalence of naturally occurring resistance in INI-naive subjects with CRF02_AG.^24–26^ The CORONET project (Common Sequence Repository and Research Network for Integrase Inhibitors) is a European network for surveillance and collaborative research on INI resistance. A common repository is produced from genotypic resistance tests performed at multiple collaborating centres, which provide care for ethnically diverse cohorts. The aim of this analysis, the first of the CORONET database, was to determine the integrase sequence profiles of patients infected with diverse HIV-1 clades that were either INI naive or experiencing viraemia while receiving raltegravir-containing ART, and identify known and novel integrase mutations associated with raltegravir selective pressure, with a specific focus on the impact of HIV-1 clade on mutation patterns.

## Methods

### Study population

Collection of integrase sequences started in 2008 from nine European laboratories and from the UK HIV Drug Resistance Database (http://128.40.115.16/hivrdb/public/default.asp) and the EuResist Database (http://www.euresist.org). Available clinical data included HIV-1 RNA load and ART status at the time of integrase sequencing. The populations undergoing sequencing were INI-naive patients and patients experiencing a viral load >50 copies/mL while receiving raltegravir-containing ART. Sanger sequencing was performed at each participating centre according to local practice and using in-house protocols. All sequences were re-analysed centrally. The current analysis is based upon all submissions made between 2008 and 2011. The Ethics Committee of the Royal Free Hospital in London approved the analysis of fully anonymous data; written informed consent was not required (06/Q0501/125 and MREC/01/2/10). Separate ethics approvals regulate the UK HIV Drug Resistance Database and the EuResist Database.

### HIV-1 clades

HIV-1 clades were assigned by submitting the integrase sequences to the Stanford University HIV Drug Resistance Database (http://hivdb.stanford.edu) and Rega HIV-1 subtyping tools (http://dbpartners.stanford.edu:8080/RegaSubtyping/stanford-hiv/typingtool/). Where results were inconclusive, the NCBI genotyping tool (http://www.ncbi.nlm.nih.gov/projects/genotyping) was used and sequences were characterized by phylogenetic analysis with PhyML. A maximum-likelihood tree was produced using a generalized time-reversible (GTR) model of nucleotide substitution with parameters estimated in PAUP* (bootstrap1000) and integrase reference sequences derived from the Los Alamos Database (http://www.hiv.lanl.gov).

### Resistance mutations

Integrase sequences were screened for INI resistance-associated mutations (RAMs) as defined by the Stanford University HIV Drug Resistance Database (February 2014) (http://hivdb.stanford.edu) and the International AIDS Society.^27^ Major RAMs comprised T66A/I/K, E92Q/G/V, F121Y, G140S/A/C, Y143H/R/C/K, S147G, Q148H/R/K and N155H/S/T. Minor RAMs comprised mutations at codons 51, 74, 95, 97, 114, 128, 138, 145, 146, 151, 153, 157, 163, 230 and 263. Cross-resistance to dolutegravir was classified according to the definition used in the VIKING clinical trials,^18^ modified to include the potential low-level resistance effects conferred by isolated Q148 mutations (in the absence of other INI RAMs) as indicated by the Stanford University HIV Drug Resistance Database. Based on available data from raltegravir-experienced patients,^18^ resistance was defined for twice-daily use of dolutegravir. Dolutegravir resistance categories were as follows: (i) no resistance, absence of Q148H/R/K; (ii) low-level resistance, Q148H/R/K in the absence of G140S/A/C, L74I and E138A/K/T; (iii) intermediate-level resistance, Q148H/R/K with one of the mutations G140S/A/C, L74I or E138A/K/T; and (iv) high-level resistance, Q148H/R/K with two or three of the mutations G140S/A/C, L74I and E138A/K/T.

### Statistical analysis

Fisher's exact test was used to compare amino acid frequencies across all integrase codons in INI-naive versus raltegravir-experienced subjects and to compare prevalence of INI RAMs in subtype B versus non-B HIV-1 clades. Viral load levels at the time of integrase sequencing were compared between different subgroups by the Wilcoxon rank-sum test. All analyses were performed using STATA version 12.1.

## Results

### Prevalence and patterns of integrase RAMs

**Figure 1.**
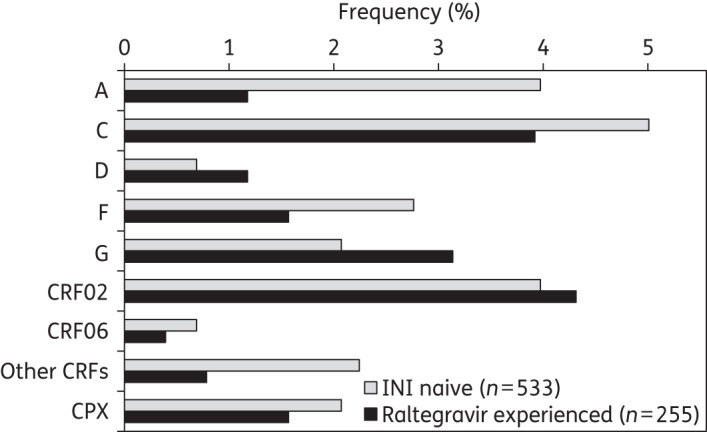
Frequency of non-B HIV-1 clades in INI-naive and raltegravir-experienced patients. Other CRFs comprised CRF01, CRF05 and CRF35.

Integrase sequences were analysed from 533 INI-naive subjects and 255 raltegravir recipients with viraemia. The most prevalent HIV-1 clades in the two groups were subtype B (75% and 82%, respectively), subtype C (5% and 4%) and CRF02_AG (4% and 4%) (Figure 1). The other non-B clades were diverse and included complex sequences (2% in each group) for which the clade could not be assigned.

Among the 533 INI-naive subjects, the median viral load at the time of sequencing was 21 476 copies/mL (IQR 2298–113 308); no patient was found to carry major INI RAMs. Among the 255 raltegravir recipients, the median viral load was 3523 copies/mL (IQR 555–27 793); 113/255 (44.3%) patients showed one or more major INI RAMs. In raltegravir recipients with versus those without major INI RAMs the median viral load was 3420 copies/mL (IQR 1195–20 899) versus 3834 copies/mL (IQR 376–32 342), respectively (*P* = 0.30). The most commonly observed major INI RAMs were N155H (57, 22.4%), G140S (33, 12.9%), Q148H (28, 11.0%), Q148R (15, 5.9%), E92Q (9, 3.5%) and Y143R (9, 3.5%) (Table 1). The most prevalent mutation profiles were N155H in isolation (45, 17.6%), Q148H/R/K + G140S/A (35, 13.7%) and Y143R/C/H in isolation (12, 4.7%). The three major resistance profiles (codons 143, 148 and 155) did not co-occur in our study. Preferential associations comprised N155H with E92Q, Y143H/R/C with T97A and Q148H/R/K with G140S/A. Conversely, mutations that were mutually exclusive comprised E92Q and T97A with Q148H/R/K, and G140S/A with Y143R/C/H and N155H. An additional four (1.6%) raltegravir recipients, all with subtype B, showed non-classic substitutions at major integrase resistance codons, comprising: (i) E92A, detected with the INI RAMs N155H and T97A (viral load 3170 copies/mL); (ii) S147I (viral load 477 copies/mL); (iii) N155D (viral load 140 copies/mL); and (iv) N155Q (viral load 111 copies/mL).

**Table 1.** Major integrase RAMs detected in raltegravir recipients with viraemia

RAMs, *n* (%)^a^						Pattern, *n* (%)^b^
E92Q, 9 (3.5)	G140S/A, 37 (14.5)	Y143R/C/H, 14 (5.5)	S147G, 1 (0.4)	Q148H/R/K, 44 (17.3)	N155H, 57 (22.4)	
					X	45 (17.6)
	X			X		35 (13.7)
		X				12 (4.7)
X					X	8 (3.1)
				X		6 (2.4)
		X			X	2 (0.8)
			X	X		1 (0.4)
				X	X	1 (0.4)
	X			X	X	1 (0.4)
	X					1 (0.4)
X						1 (0.4)

^a^Overall 113/255 (44.3%) raltegravir recipients had one or more major RAMs. The mutations observed were: E92Q (*n* = 9); G140S (*n* = 33) and G140A (*n* = 4); Y143R (*n* = 9), Y143C (*n* = 4) and Y143H (*n* = 1); Q148H (*n* = 28), Q148R (*n* = 15) and Q148K (*n* = 1); and N155H (*n* = 57).

^b^X symbols within each row indicate mutations present in each pattern.

Minor INI RAMs that occurred at a frequency of >2% in raltegravir recipients are shown in Table 2. Of these, L74M, T97A, E138K, V151I and G163R were significantly associated (*P* < 0.01) with raltegravir exposure.

**Table 2.** Minor integrase mutations observed to occur at a frequency of >2% in raltegravir recipients with viraemia

Mutation	Frequency, *n* (%)		*P*	Positive association with major INI RAMs^a^
	raltegravir naive (*n* = 533)	raltegravir experienced (*n* = 255)		
74I	35 (6.6)	13 (5.1)	0.53	
74M	3 (0.6)	8 (3.1)	0.007	
97A	8 (1.5)	20 (7.8)	<0.001	Y143R/C/H
138K	0 (0.0)	9 (3.5)	<0.001	Q148H/R/K
151I	10 (1.9)	25 (9.8)	<0.001	N155H
157Q	11 (2.1)	6 (2.4)	0.80	
163R	3 (0.6)	10 (3.9)	0.001	
163E	29 (5.4)	7 (2.7)	0.10	
230N	51 (9.6)	17 (6.7)	0.22	

^a^Positive association indicated preferential co-occurrence with major INI RAMs.

### Novel mutations associated with raltegravir exposure

Comparing sequences from INI-naive and raltegravir-experienced patients, three integrase positions were newly identified statistically as being subjected to selective pressure, involving codons 159 (*P* = 0.009), 161 (*P* = 0.004) and 170 (*P* = 0.009). These mutations occurred in subtype B (K159Q/R, I161L/M/N/T/V and E170A/G) and CRF02_AG (I161L/T). They occurred commonly alongside other mutations, e.g. K159R + I161L occurred with Q148H + G140S, and E170A occurred with N155H and Q148H + G140S (Table S1, available as Supplementary data at *JAC* Online). In addition, the analysis revealed that raltegravir selective pressure was associated with significantly reduced diversity at integrase positions 24 and 167 (*P* < 0.001 for both).

### Influence of HIV-1 clade on integrase mutational profiles

Comparing subtype B with non-B clades, mutations at codons 148 and 140 occurred in 42 (20.1%) versus 2 (4.3%) (*P* = 0.009) and 36 (17.2%) versus 1 (2.2%) (*P* = 0.005), respectively (Table 3). The most prevalent mutations at these positions—G140S and Q148H—were exclusively found in subtype B sequences. G140A occurred in 3 subtype B sequences and 1 subtype G sequence, and Q148R occurred in 13 subtype B sequences, 1 subtype C sequence and 1 subtype G sequence. Conversely, E92Q was significantly less frequent in subtype B (4/209, 1.9%) than in non-B clades (5/46, 10.9%) (*P* = 0.02), occurring in subtypes C (*n* = 2), G (*n* = 1), A (*n* = 1) and CRF09 (*n* = 1). In patients with one or more major RAMs, the median viral load at the time of sampling was 3535 copies/mL (IQR 1472–25 704) versus 1820 (IQR 555–11 660) in subjects with B versus non-B clades, respectively (*P* = 0.33).

**Table 3.** Major integrase RAMs in raltegravir recipients at positions that differ in mutation frequency between subtype B and non-B clades

RAM	Subtype B (*n* = 209)	Non-B clades (*n* = 46)
E92Q, *n* (%)	4 (1.9)	5 (10.9) [A, C, G, CRF09]^a^
G140S, *n* (%)	33 (15.8)	0 (0)
G140A, *n* (%)	3 (1.4)	1 (2.2) [G]^a^
Q148H, *n* (%)	28 (13.4)	0 (0)
Q148R, *n* (%)	13 (6.2)	2 (4.3) [C, G]^a^
Q148K, *n* (%)	1 (0.5)	0 (0)

^a^Non-B clades with the mutation are indicated in square brackets.

Codon usage in integrase sequences of INI-naive patients was examined in order to define its relation to the mutation profiles observed in raltegravir-experienced patients (Table 4). Sequences differed by clade in their propensity to acquire mutations at codon 140. Overall 384/399 (96%) subtype B sequences had GGT or GGC (or a mixture of both) at position 140, requiring a single nucleotide substitution to replace glycine with serine (AGT or AGC). The remaining 4% of subtype B sequences showed GGA, GGM, GGK and GGW. In contrast, all 134 (100%) non-B sequences had GGA or GGG (or a mixture of both), requiring more than one nucleotide substitution to encode serine. There was no consistent difference in codon usage at positions E92 and Q148.

**Table 4.** Codon usage in INI-naive subjects at positions showing a different mutation frequency between subtype B and non-B clades after raltegravir treatment

Position	Subtype B (*n* = 399)		Non-B clades (*n* = 134)	
	triplet	*n* (%)	triplet	*n* (%)
92	GAG	248 (62.2)	GAG	7 (5.2)
	GAA	117 (29.3)	GAA	123 (91.8)
	GAR	34 (8.5)	GAR	3 (2.2)
140	GGC	331 (83.0)	GGA	84 (62.7)
	GGT	35 (8.8)	GGG	43 (32.1)
	GGY	18 (4.5)	GGR	7 (5.2)
	other	15 (3.8)		
148	CAA	378 (94.7)	CAA	106 (79.1)
	CAG	13 (3.3)	CAG	27 (20.1)

### Cross-resistance to dolutegravir

**Figure 2.**
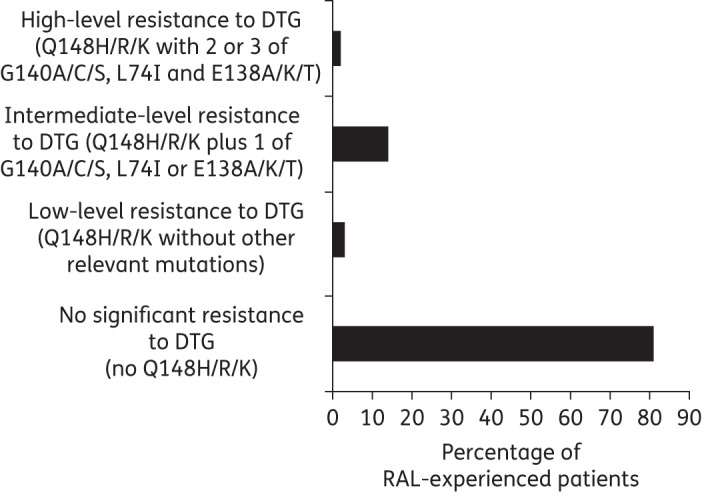
Predicted cross-resistance to dolutegravir (twice-daily dosing) in raltegravir recipients with viraemia. DTG, dolutegravir; RAL, raltegravir.

Of the 255 raltegravir recipients, 211 (83%) had no predicted resistance to dolutegravir (for twice-daily use). Among those with any predicted dolutegravir resistance, 4 (1.6%) had low-level resistance, 36 (14.1%) had intermediate-level resistance and 4 (1.6%) had high-level resistance (Figure 2). Reflecting the lower prevalence of the Q148H/R/K + G140A/S pathway, the prevalence of predicted dolutegravir cross-resistance among subjects with non-B clades was significantly lower than in subjects with subtype B, both overall (2/46, 4.3% versus 42/209, 20.1%; *P* = 0.009) and when restricting the comparison to subjects with intermediate- to high-level dolutegravir cross-resistance (1/46, 2.2% versus 39/209, 18.7%; *P* = 0.003).

## Discussion

In this large European cohort with diverse HIV-1 clades, there was no evidence of major INI RAMs among INI-naive subjects. In contrast, nearly half of patients experiencing viraemia on raltegravir-containing ART showed one or more major INI RAMs. The prevalence of genotypic INI resistance measured in raltegravir recipients with virological failure was consistent with the resistance rates reported from clinical trials (32%–68%).^2,6^ Similar rates were reported from an observational cohort, which also found an association between low plasma raltegravir concentrations (taken to indicate poor adherence) and lack of detectable INI RAMs at virological failure.^28,29^ While we did not have raltegravir concentrations, we did not observe a significant difference in viral load levels between subjects with and without detectable INI RAMs, which might have indirectly suggested a difference in adherence levels.

The three major recognized pathways of genotypic resistance to raltegravir (N155H, Q148H/R/K and Y143R/C/H) were represented in the raltegravir-experienced population. Interestingly, co-occurrence of these major pathways was not seen, possibly representing functional incompatibility of all three pathways in the context of a single virus. Indeed, the N155H and Q148H/R/K pathways have been shown to sequentially emerge in separate genomes within a given individual over time.^30^ Consistent with available data, there was a strong preferential association of N155H with E92Q, Y143H/R/C with T97A, and G140S/A with Q148H/R/K. Of these three mutually antagonistic genotypic patterns, Q148H/R/K and G140S/A were significantly more prevalent in subtype B than in non-B clades, occurring only in three subjects (with subtype C and subtype G). Strikingly, Q148H and G140S occurred in 13% and 16% of subtype B sequences, respectively, but were absent from all non-B clades analysed. This finding could be wholly explained through differences in codon usage between subtype B and non-B clades in INI-naive patients. In the vast majority of subtype B sequences, one nucleotide substitution was required to replace glycine with serine at integrase codon 140. In all the non-B clades examined (A, C, D, F, G, CRF01, CRF02, CRF06, CRF35_AD and several complex mosaic sequences) two nucleotide substitutions were required, thus raising the genetic barrier to the emergence of G140 mutants. As mutations at codon 140 play a key role in restoring the fitness of Q148 mutants,^31^ their occurrence can also influence the emergence of Q148H/R/K, thus explaining the reduced prevalence of Q148 mutants observed in non-B subtypes. Small studies previously proposed a higher genetic barrier for G140S and G140C in subtypes C, CRF01 and CRF02^24–26,32^ and more recently a large study of INI-experienced patients identified more Q148 mutations in subtype B versus non-B subtypes.^8^ Our findings thus confirm and extend these previous observations, indicating the unique propensity of subtype B to the development of the Q148 + G140 mutation pathway. Taken together, these findings show that theoretical predictions based on assessment of the genetic barrier to resistance translate into the actual acquisition of INI mutations in contemporary clinical practice.

Notably, the Q148 mutation pathway confers the highest level of resistance to available INIs, including a significant level of cross-resistance to dolutegravir.^33^ *In vitro* studies show that mutations at codons Y143 and N155 confer EC_50_ fold changes for dolutegravir consistently <3.^14,15^ Variable levels of resistance are seen with Q148 mutations depending on the presence of other INI RAMs.^14,15^ In the Phase 3 VIKING-3 trial, INI-experienced patients showed different virological responses to dolutegravir twice daily plus an optimized background regimen depending on the baseline mutation pattern.^18^ Virological suppression <50 copies/mL at 24 weeks was 100/126 (79%) in the absence of baseline Q148 mutations (regardless of the presence of Y143 and N155 mutants), 21/36 (58%) with Q148 mutations plus one minor INI RAM and 5/21 (24%) in the presence of Q148 plus at least two minor INI RAMs.^18^ Our findings are therefore of practical importance in relation to the likelihood of cross-resistance to dolutegravir in raltegravir-experienced patients. Q148H/R/K mutations were present in 17% of raltegravir-experienced patients and ∼16% of the raltegravir-experienced patients were classified as having intermediate- to high-level resistance to dolutegravir. These data are reassuring as they indicate that most raltegravir-experienced patients, including the large majority of subjects with non-B clades, will retain at least partial susceptibility to twice-daily dolutegravir.^18^ As clinical experience with dolutegravir in INI-experienced patients remains limited, a role for other mutation profiles in conferring dolutegravir cross-resistance cannot be excluded.

In addition to recognized INI RAMs, non-classic amino acid substitutions at the major integrase resistance codons 92, 147 and 155 were detected in four raltegravir recipients with subtype B. The analysis also identified novel mutations as significantly associated with raltegravir selective pressure—K159Q/R, I161L/M/N/T/V and E170AG. Studies employing site-directed mutagenesis are necessary to investigate the phenotypic properties of these novel mutations and confirm a role in INI resistance. It should be noted that our observations do not exclude a role in resistance for rarer variants that would not be captured by a stringent statistical approach.^34^

The lack of major INI RAMs in INI-naive subjects suggests that transmitted INI resistance was rare in 2008–11. Although transmitted INI RAMs are still uncommonly described even among subjects with primary HIV infection,^35–42^ the risk may increase with expanded use of INIs, indicating the need for ongoing surveillance. Importantly, the data also indicate that in the absence of INI selective pressure, major INI RAMs do not occur spontaneously in the diverse non-B clades analysed. Others have reported the presence of major INI RAMs in INI-naive patients infected with subtype CRF02_AG in a small cohort in Cameroon.^26^ We did not find evidence of major INI RAMs in 22 INI-naive patients infected with CRF02_AG in our study.

The study illustrates the ongoing importance of collaborative research between different centres in order to collect and meaningfully interpret data from large numbers of patients in routine care, and we hope that this study will prove useful in designing analyses of large depositories such as the Stanford dataset. However, the study has limitations. Policies and methods for resistance testing are likely to have differed across centres. The full treatment history and the duration of viraemia prior to the resistance test were not available and caution is therefore required in the interpretation of resistance rates. In subtype B, it has been shown that N155 mutants tend to be replaced by the more resistant and fitter Q148 + G140 variants with ongoing drug selective pressure. Thus, the resilience of non-B clades with respect to the emergence of these mutations should be considered relative and time-dependent rather than absolute.

## Funding

The study was supported by a research award made by Merck to the Royal Free Charitable Trust. The funder approved the study design and had no involvement in the conduct of the study, data collection and analysis, and preparation of the manuscript. T. D. is a Wellcome Trust Research Training Fellow.

## Transparency declarations

None to declare.

## Supplementary data

Table S1 is available as Supplementary data at *JAC* Online (http://jac.oxfordjournals.org/).

Supplementary Data
