# Supplementary material for: Integrase inhibitor (INI) genotypic resistance in treatment-naive and raltegravir-experienced patients infected with diverse HIV-1 clades
Source: J Antimicrob Chemother. 2015 Aug 26;70(11):3080–6. doi: 10.1093/jac/dkv243 (PMC4613743; doi:10.1093/jac/dkv243)
Supplement: Supplementary Data [file supp_70_11_3080__index.html]

Integrase inhibitor (INI) genotypic resistance in treatment-naive and raltegravir-experienced patients infected with diverse HIV-1 clades — Integrase inhibitor (INI) genotypic resistance in treatment-naive and raltegravir-experienced patients infected with diverse HIV-1 clades — Supplementary Data 

# Integrase inhibitor (INI) genotypic resistance in treatment-naive and raltegravir-experienced patients infected with diverse HIV-1 clades

## Supplementary Data

Supplementary Data

- Supplementary Data - Docx file
