# Supplementary material for: Integrase inhibitor (INI) genotypic resistance in treatment-naive and raltegravir-experienced patients infected with diverse HIV-1 clades
Source: J Antimicrob Chemother. 2015 Aug 26;70(11):3080–6. doi: 10.1093/jac/dkv243 (PMC4613743; doi:10.1093/jac/dkv243)
Supplement: Supplementary Data [file supp_dkv243_dkv243supp.docx]

**Supplementary data**

**Table S1.** Novel integrase mutations associated with raltegravir exposure

| Novel mutation | HIV-1  clade | HIV-1 RNA copies/ml | Other mutations^a^ |
| --- | --- | --- | --- |
| K159Q + I161T | B | 1413 |  |
| K159R + I161L | B | 25704 | **Q148H, G140S** |
| K159R + I161M | B | 650 |  |
| K159R + E170G | B | 140 | N155D |
| I161V | B | 114 |  |
| I161L | CRF02 | 3800 |  |
| I161N | B | 111 | N155Q, E157K |
| E170A | B | 436 | **N155H** |
| E170A | B | 4898 | L74M, V151I |
| E170A | B | 15400 | **Q148H, G140S** |

^a^Major integrase inhibitor resistance-associated mutations in bold
